# Clinical Forms of Chikungunya Virus Infection: The Challenge and Utility of a Consensus Definition

**DOI:** 10.4269/ajtmh.18-0468a

**Published:** 2018-08

**Authors:** Moustapha Dramé, Lukshe Kanagaratnam, Maxime Hentzien, Jean-Luc Fanon, Seendy Bartholet, Lidvine Godaert

**Affiliations:** Faculty of Medicine, University of Reims Champagne-Ardenne, Reims, France; Department of Research and Public Health, Robert Debré Hospital, University Hospital of Reims, Reims, France; E-mails: mdrame@chu-reims.fr and lkanagaratnam@chu-reims.fr; Faculty of Medicine, University of Reims Champagne-Ardenne, Reims, France; Department of Infectiology, Robert Debré Hospital, University Hospital of Reims, Reims, France; E-mail: mhentzien@chu-reims.fr; Department of Geriatrics, University Hospitals of Martinique, Martinique, France; E-mails: jean-luc.fanon@chu-martinique.fr, seendy.bartholet@chu-martinique.fr, and lidvine-michele.godaert-simon@chu-martinique.fr

Dear Sir,

We read with great interest the recently published article by Dorléans et al.^1^ entitled “*Outbreak of Chikungunya in the French Caribbean Islands of Martinique and Guadeloupe*.”

The authors provide useful and relevant data about this emerging disease, and the study had several strong points that deserve to be underlined. First, data collection was prospective, using a standardized questionnaire and, thus, of high quality. Second, cases of chikungunya virus infection (CVI) were prospectively identified by reverse transcriptase-polymerase chain reaction (RT-PCR) or by serology immunoglobulin M (IgM). Third, preliminary data were recorded using a specialized informatics system dedicated to priority infectious diseases in France. Fourth, additional data were retrospectively recorded by specialized research personnel. However, we would like to make some additional remarks specifically regarding the population of patients aged 65 years or older.

In 2015, the World Health Organization (WHO) brought together an expert group to develop consensus definitions of the clinical forms of CVI. The resulting definitions^2^ described three clinical forms at the acute phase, based on clinical, epidemiological, and laboratory criteria. A confirmed typical case is defined by “fever AND joint pain with acute onset” AND “residing or visiting areas with local transmission of Chikungunya” OR “laboratory confirmation by immunoglobulin or RT-PCR.”^2^ Confirmed atypical cases are defined by the same criteria AND the presence of other clinical or biological manifestations (including neurological, cardiovascular, and hepatic findings). Confirmed severe cases are defined by the same criteria and dysfunction of at least one organ or system that threatens life and requires hospitalization. In their article, Dorléans et al.^1^ classified their patients according to three clinical forms (typical, atypical, and severe) based on signs observed at the acute phase but without reference to the WHO classification. Indeed, the authors used the same terms as those described by the WHO, but the criteria are not the same, which could create confusion and may leave room for misinterpretation.

For example, according to the definition proposed by Dorléans et al.,^1^ tenosynovitis, rash, or diarrhea would each alone be considered as representative of typical CVI. Similarly, encephalitis, which can be life-threatening, was considered as a sign of atypical presentation, and not as severe disease. Several authors have already reported that the definition of the clinical forms of CVI is not applicable in infants^3^ or in older adults.^4^ In the study reported by Dorléans et al.,^1^ 17% of subjects were aged less than 1 year and 40% were aged more than 60 years. Therefore, this raises the question of the validity of “one size fits all” definitions supposedly applicable to all populations.

Regarding elderly subjects, the frequently atypical clinical profile of CVI in this population renders it difficult to unmask the diagnosis.^4^ Accordingly, Godaert et al.^5^ have reported that the rate of under-diagnosis of CVI in a population of patients attending the emergency department was more than 20% among those aged 65 years or more, compared with around 3% in younger patients. Indeed, the usual presentation of CVI is different in elderly subjects,^4^ and the screening tools used in 2014 perform only moderately well in elderly subjects.^6^ In view of the inclusion criteria, and in particular, the hospital-based population included in the study by Dorléans et al.,^1^ the frequency of each form of disease described in their article likely does not represent the true frequency observed in the general population.

The authors underline that subjects aged 60 years and older represented 69.5% of patients with severe forms versus 33.6% of non-severe forms of CVI. The fact that severe forms of CVI are more common in hospitalized older adults has also been reported previously.^7^ Godaert et al.^4^ compared the frequency of clinical forms, as defined by the WHO, between subjects aged 65 years and older and their younger counterparts, among patients presenting from their home to the emergency department within 3 days of onset of symptoms of CVI. There was no statistically significant difference in the frequency of severe disease between the two populations, but older subjects more frequently presented atypical forms, as defined by the WHO. This finding suggests that there may be some selection bias among hospitalized patients.

Regarding in-hospital mortality, Dorléans et al.^1^ report a death rate of 4% in the overall population. A previous study among patients aged 65 years and older reported a mortality of 9.1% in this group, and the predictors of death identified were the presence of cardiovascular, respiratory, neurological, or digestive disorders, and a history of alcoholism.^8^ Therefore, these should all be considered as signs of disease severity in this population and not signs of atypical presentation. Dorléans et al.^1^ reported that there were 74 deaths in their study, but it would have been interesting to specify the distribution of these deaths according to the clinical forms they described.

All these points underline the importance of using consensus definitions for the clinical forms of disease that are applicable in each patient population. The use of standardized terms would greatly facilitate comparisons between studies. Secondly, the findings of Dorléans et al.^1^ underscore the fact that CVI takes a heavy toll on older subjects and, therefore, specific studies are warranted to improve diagnosis and provide adequate management in this population.
